# Polyp Matching in Colon Capsule Endoscopy: Pioneering CCE-Colonoscopy Integration Towards an AI-Driven Future

**DOI:** 10.3390/jcm13237034

**Published:** 2024-11-21

**Authors:** Ian Io Lei, Ramesh Arasaradnam, Anastasios Koulaouzidis

**Affiliations:** 1 Institute of Precision Diagnostics & Translational Medicine, University Hospital of Coventry and Warwickshire, Clifford Bridge Rd, Coventry CV2 2DX, UK; r.arasaradnam@warwick.ac.uk; 2 Warwick Medical School, University of Warwick, Coventry CV4 7AL, UK; 3 Department of Digestive Diseases, University Hospitals of Leicester NHS Trust, Leicester LE1 5WW, UK; 4 Leicester Cancer Centre, University of Leicester, Leicester LE1 7RH, UK; 5 Surgical Research Unit, Odense University Hospital, 5700 Svendborg, Denmark; akoulaouzidis@hotmail.com; 6 Department of Surgery, OUH Svendborg Sygehus, 5700 Svendborg, Denmark; 7 Department of Clinical Research, University of Southern Denmark, 5230 Odense, Denmark; 8 Department of Gastroenterology, Pomeranian Medical University, 70-204 Szczecin, Poland

**Keywords:** colon capsule endoscopy, capsule endoscopy, polyp, colorectal cancer, colon cancer, colonoscopy

## Abstract

**Background**: Colon capsule endoscopy (CCE) is becoming more widely available across Europe, but its uptake is slow due to the need for follow-up colonoscopy for therapeutic procedures and biopsies, which impacts its cost-effectiveness. One of the major factors driving the conversion to colonoscopy is the detection of excess polyps in CCE that cannot be matched during subsequent colonoscopy. The capsule’s rocking motion, which can lead to duplicate reporting of the same polyp when viewed from different angles, is likely a key contributor. **Objectives**: This review aims to explore the types of polyp matching reported in the literature, assess matching techniques and matching accuracy, and evaluate the development of machine learning models to improve polyp matching in CCE and subsequent colonoscopy. **Methods**: A systematic literature search was conducted in EMBASE, MEDLINE, and PubMed. Due to the scarcity of research in this area, the search encompassed clinical trials, observational studies, reviews, case series, and editorial letters. Three directly related studies were included, and ten indirectly related studies were included for review. **Results**: Polyp matching in colon capsule endoscopy still needs to be developed, with only one study focused on creating criteria to match polyps within the same CCE video. Another study established that experienced CCE readers have greater accuracy, reducing interobserver variability. A machine learning algorithm was developed in one study to match polyps between initial CCE and subsequent colonoscopy. Only around 50% of polyps were successfully matched, requiring further optimisation. As Artificial Intelligence (AI) algorithms advance in CCE polyp detection, the risk of duplicate reporting may increase when clinicians are presented with polyp images or timestamps, potentially complicating the transition to AI-assisted CCE reading in the future. **Conclusions**: Polyp matching in CCE is a developing field with considerable challenges, especially in matching polyps within the same video. Although AI shows potential for decent accuracy, more research is needed to refine these techniques and make CCE a more reliable, non-invasive alternative to complement conventional colonoscopy for lower GI investigations.

## 1. Background

Colon capsule endoscopy (CCE) is becoming widely available in Europe as an alternative to conventional colonoscopy for investigating lower gastrointestinal (GI) symptoms. It is a dual-camera capsule that offers a 344° field of view with a battery life of 12 h to capture video of most of the small bowel and the whole colon [1]. With its latest model, PillCam Crohn’s Capsule (Medtronic, Dublin, Ireland), a panenteric capsule that is capable of comprehensively examining the whole of small and large bowels, this technology has an extra edge compared to colonoscopy, which limits to terminal ileum and colon only [2].

Its other key advantages include being non-invasive, sedation-free, radiation-free, and more comfortable, with no risk of perforation during the procedure and the potential of home delivery service [3]. Despite these benefits, its uptake has yet to meet expectations, primarily due to one major drawback: the need for follow-up conventional endoscopy. This major drawback is influenced by three predominant factors: the need for excellent bowel preparation, incomplete procedures, and the detection of pathologies that require therapeutic intervention or biopsy. Among these, the issue of incomplete procedures (where the capsule’s battery is exhausted before excretion) has been partially mitigated with the introduction of prucalopride, which shortens capsule transit time [4]. However, an enhanced bowel preparation regimen for CCE is unavoidable, especially when CCE cannot wash or suction the stool nor position itself to better view the area of interest (see Table 1) [5].

Even with adequate bowel preparation, accurate pathology identification remains a critical factor driving the need for conversion from CCE to traditional colonoscopy, limiting CCE’s potential as a widely accepted standard tool alongside colonoscopy. A significant issue is the duplication of polyp reporting, where the same polyp may be mistakenly counted multiple times, further complicating clinical interpretation and follow-up requirements. This leads to an overestimation of polyp numbers. According to the European Society of GI Endoscopy (ESGE) guidelines, polyp count is one of the two critical criteria used to determine the need for further conventional endoscopy [6]. Therefore, this duplication can result in unnecessary or potentially deferrable colonoscopies, undermining CCE’s cost-efficiency and leading to patient dissatisfaction when they must repeat the uncomfortable bowel preparation for a second procedure.

**Table 1:** The Comparison between CCE and ileocolonoscopy.

	Colon Capsule Endoscopy (CCE)/Panenteric capsule endoscopy (PCE) 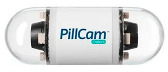	Ileocolonoscopy 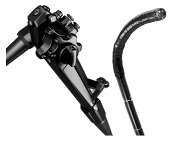
Extent of Gastrointestinal tract examined	Gastic antrum, small bowel and colon	Terminal ileum and colon only
Patient Safety	Non-invasive with minimal capsule retention risk, reliant on patient selection (0.73–2%) [7]	Invasive with perforation risk: 88 per 100,000 people (0.088%) [8]
Bowel preparation requirement	Additional Low residue diet or high volume of laxative e.g., Polyethylene Glycol (PEG) in addition to standard bowel preparation [9]	Standard bowel preparation including low volume bowel preparation [10]
Ability in taking biopsies and therapy	Unable to take biopsies or perform therapeutics	Able to take biopsies or perform therapeutics
Localisation	No scope guide for localisation of pathology other than visual landmarks such as ICV, appendiceal orifice and anal cushion [5]	Scope guide is available for more accurate localisation of the pathologies within the colon [11]
Procedure time	Average reading time: 45–60 min [12]	Average 30 min procedural slots [13]
Incomplete procedure	Range from 68% to 100% [9]	>90% caecal intubation in routine and >95% in screening colonoscopy [14]
Sedation requirement	No sedation	Sedation might be required
Patient discomfort	Painless [15]	Discomfort and pain are primary concerns for patients [16]
Booster laxative requirement	Yes	No
Home Delivery Service	Yes [17]	No

Images used with permission: PillCam Colon 2 (source: https://www.medtronic.com/en-us/healthcare-professionals/products/digestive-gastrointestinal/capsule-endoscopy/endoscopy-systems/pillcam-colon-2-system.html, accessed on 17 November 2024) and Olympus Colonoscope (source: https://www.olympus.co.uk/medical/en/Products-and-solutions/Medical-specialities/Gastroenterology/, accessed on 17 November 2024).

In addition, the differences between polyps identified in the initial CCE and those found during subsequent conventional endoscopy can cause significant anxiety for both patients and clinicians beyond just the economic costs. This often leads to concerns about whether the endoscopist might have missed polyps, potentially leaving behind lesions that could develop into cancer. These concerns are understandable, as colonoscopy has flaws despite being considered the gold standard. As reported in the literature, polyp miss rates range from 10% to 24% [18,19].

The discrepancy in identifying polyps during colonoscopy is partly due to missed polyps and false positives, such as duplicate reporting and the misidentification of mucosal folds as polyps. Another issue is that the polyps identified in colonoscopy may not accurately match those identified in the prior CCE. To address this, it is essential to focus on two critical stages of polyp matching: first, matching polyps within the same CCE video to avoid duplicate reporting, and second, matching polyps between the initial CCE video and the subsequent colonoscopy. Polyp matching within CCE has historically been underdeveloped and often overlooked, mainly because it is an intuitive process clinicians perform while reviewing the CCE video. This step is seamlessly integrated into the reporting process and has yet to be a significant focus in CCE analysis. However, with the integration of AI, polyp matching is poised to become a crucial aspect of CCE workflows. As AI systems are developed to identify and highlight only relevant images or thumbnails with detected pathology, they will primarily serve in Computer-Assisted Detection (CADe) rather than full Computer-Assisted Diagnosis (CADx), focusing on pathology detection rather than polyp characterisation or diagnosis. This shift implies that clinicians’ roles will transition from polyp detection from a full video review to primarily polyp matching, characterisation, and diagnosis, enhancing efficiency much like the mechanisation seen during the 19th-century Industrial Revolution.

In anticipation of this AI-driven future, this review explores the two primary methods of polyp matching reported in the literature, assesses clinician accuracy, and examines the development of machine learning models for polyp matching within CCE and for CCE-to-colonoscopy comparisons to improve diagnostic accuracy and workflow efficiency.

## 2. Methods

A systematic search was conducted based on PRISMA recommendations to retrieve published review articles, editorial letters, and clinical trials addressing polyp matching within CCE or between initial CCE and subsequent colonoscopy [20,21]. The primary aim was to identify techniques or approaches proposed for polyp matching within the same CCE video or between CCE and colonoscopy, focusing on their effectiveness in avoiding double reporting and reducing the false positive rate, with colonoscopy as the gold standard. A secondary outcome was to assess Artificial Intelligence (AI) applications in improving polyp matching, either within CCE or between CCE and subsequent colonoscopy.

### 2.1. Eligibility Criteria

Given the limited research in this niche area of CCE, the search included all clinical and prospective trials, observational studies, qualitative studies, reviews, case series, and editorial letters that evaluated polyp detection and polyp matching within CCE or compared CCE to subsequent conventional colonoscopy. The search was restricted to English-language studies only. Due to the scarcity of papers addressing polyp matching, the search terms focused broadly on polyps in both CCE and colonoscopy, including studies involving adult and paediatric populations. A straightforward comparison of polyps within the same CCE video or with conventional colonoscopy as the reference standard was required. No restrictions were placed on the recruitment criteria of CCE patients within the included studies. Conference abstracts were excluded due to their high risk of bias [22].

### 2.2. Information Sources

The databases that identified relevant publications included EMBASE, MEDLINE, and PubMed. Additionally, references from the extracted studies were hand-searched to identify further relevant publications. The electronic search encompassed all studies up to 1st September 2024, with no additional time restrictions. The search strategy included both MeSH and non-MeSH terms, such as “polypadenoma” and “lateral spreading tumour” (see Appendix A, Table A1). The specific search strings for each database are provided in Appendix A. Grey literature and unpublished studies were excluded from this review.

### 2.3. Study Selection

One of the authors (I.I.L.) reviewed the titles and abstracts of all retrieved studies; studies that did not meet the eligibility criteria were excluded. The inclusion criteria for the full-text review were as follows:

- Comparison in polyp detection between CCE (including CCE1, CCE2, or Crohn’s capsule) and conventional colonoscopy as the comparator.
- Polyp matching within the CCE itself or between CCE and subsequent colonoscopy.
- Evaluation of the reasons for the false positive or false negative polyp detection rates of CCE compared to colonoscopy.
- Evaluation of the reason for any intra-observer or interobserver variability in polyp detection within the same CCE video.
- Both prospective and retrospective clinical studies use the detection or diagnosis of polypoidal disease as the predominant endpoint of the study.
- Both quantitative and qualitative studies were included.

**Figure A1:**
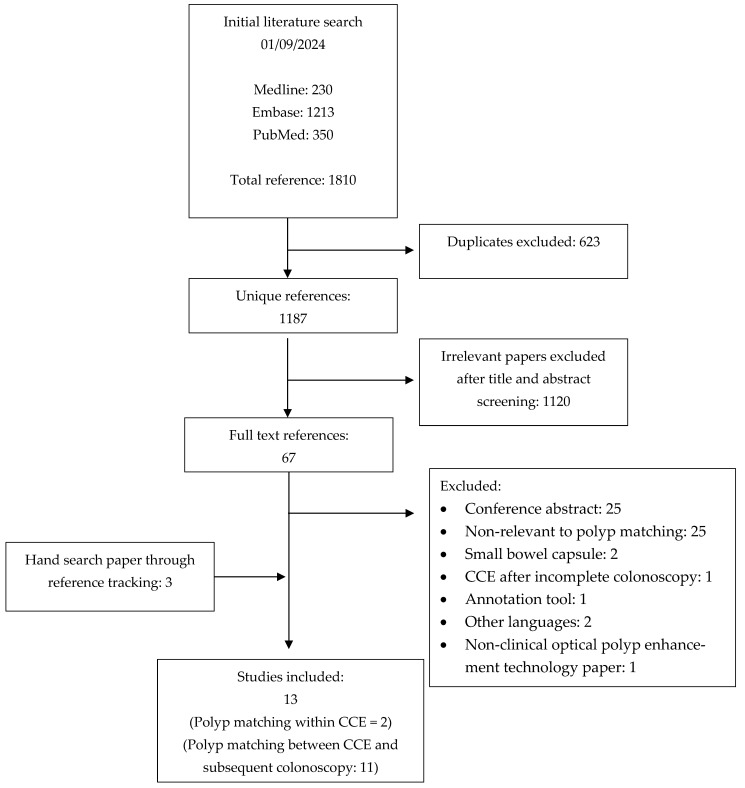
The PRISMA flowchart for systematic search.

Exclusion criteria included studies focusing predominantly on inflammatory bowel disease, CCE following incomplete colonoscopy, haemorrhoids, diverticular disease, angiodyplasia, other colitis, other comparators such as computed tomography colonography or Magnetic Resonance Imaging (MRI) and the use of small bowel capsules (see Appendix A, Figure A1).

### 2.4. Data Compilation

The final selection of studies was reviewed, followed by data extraction. The extracted details included the study type, type of polyp matching, false negative rate, false positive rate, correlation coefficient and the relevant qualitative techniques or information on polyp matching described in the study.

### 2.5. Risk of Bias

The Quality Appraisal for Diverse Studies (QuADS) version 1 tool was used to assess bias, as our review includes both qualitative and quantitative studies. Study quality was calculated as a percentage of the total score across 13 criteria, including research concept, study aims, study design, target population, sampling strategy, recruitment strategy, data collection, the format of data collection tool, data collection procedure, Analytic method, the relevance of analysis to study aim, research stakeholders’ consideration, strength and limitations. QuADS does not include a standardised cut-off score to categorise studies as high or low quality; any cut-off would be arbitrary and inconsistent with the tool’s intent. Instead, we focused on evaluating each criterion individually across studies rather than relying solely on overall scores (see Appendix A, Table A2) [23].

### 2.6. Data Synthesis and Analysis

We analysed studies on polyp matching within the same CCE video that reported false positive rates, specifically those arising from misdiagnosis or duplicate polyp reporting after a second review by an independent reader or expert panel. Relevant data was extracted, and a meta-analysis will be conducted only if a minimum of 10 studies report false positive rates related to duplicate polyp reporting. Also, studies describing any qualitative technical methods used or applied for polyp matching within the same CCE video or between CCE and subsequent colonoscopy were recorded. The false positive rate of CCE compared to colonoscopy was excluded due to the risk of missed polyps in the gold standard colonoscopy, which could lead to false negative results.

## 3. Results

Following the systematic review, only three studies were found to be directly related to polyp matching. Two focused on polyp matching within the same CCE video, while one examined polyp matching between the initial CCE video and subsequent colonoscopy (see Table 2). In the context of polyp matching within the same CCE video, one study indirectly addressed polyp number discrepancies among expert and non-expert readers of CCE, which could be attributed to double reporting and missed polyps. It also demonstrated that experienced readers had better interobserver agreement, reducing these errors. However, it did not explore the rationale behind the discrepancy in the number of polyps identified in detail [24]. Another study conducted by our group focused on criteria for polyp matching (CCE PM criterion) within the same CCE video [25]. Due to an insufficient number of identified studies, a meta-analysis was not performed. The only study that directly applied AI to polyp matching was by Blanes-Vidal et al. His research group pioneered the development of AI models for detecting polypoid lesions in CCE and matching them to polyps identified during subsequent colonoscopy [26]. Additionally, ten other studies indirectly compare polyp accuracy and differences in polyp characterisation between CCE and subsequent colonoscopy in the literature, as shown in Appendix A, Table A3.

Regarding the risk of bias, the three selected direct studies demonstrated moderate bias, with overall scores ranging from 64% to 77%. A notable limitation across studies was the lack of clarity in recruitment strategies; details on how experts were recruited were often absent, and recruitment was generally restricted to local centres or primarily within Europe. CCE expertise was defined by a minimum experience threshold, which may be insufficient for the study purpose. Additionally, patient sampling criteria were often underreported. Another limitation was the narrow consideration of research stakeholders. In the polyp matching studies, only CCE readers were involved, whereas input from expert colonoscopists or capsule nurses could have provided a broader range of perspectives and insights.

## 4. Discussion

### 4.1. Polyp Matching Within the Same CCE Video

Buijs et al. found moderate interobserver agreement in polyp reporting, with an intraclass correlation coefficient (ICC) of 0.67 for polyp number, likely due to either missed or double reporting. The ICC only improved to 0.7 among experts, highlighting the persistent risk of missed polyps and the misidentification of mucosal folds as polyps [24]. Additionally, some disagreements may be attributed to the duplicate reporting of the same polyp, which can occur when the capsule moves backwards through a previous colonic segment, capturing the same polyp multiple times [27]. These findings emphasised the continuous need for improved polyp detection through better training and reduced double reporting risk.

In clinical practice, assessing two similar polyps to avoid duplicate reporting is often intuitive and subjective, sometimes leading to inconsistency and inaccuracy. To address this issue more objectively, we developed a criterion based on available evidence and expert consensus using the RAND process, a modified Delphi process. This approach offers a more standardised method for evaluating polyps and minimising duplicate reporting. The CCE Polyp Matching criteria are outlined in Table 3 [25].

This criterion consists of 8 components that influence how two polyp images are matched within the same CCE video. If five out of the eight components are scored, the polyps are most likely considered to be the same. Each of these eight components is given equal weighting to simplify the application of the matching process.

#### 4.1.1. Intra-Polyp Characteristics for Polyp Matching

When matching polyps, the first step is to determine whether they visually resemble each other. Instead of relying solely on intuition, there are objective components to consider, such as polyp size, vascular pattern, morphology, surface characteristics, and contour. The more distinctive these features are, the easier it is to match the polyps. Agreed-upon distinctive features include pedunculated, flat, lateral spreading, and malignant appearances. Larger, advanced polyps (>10 mm) are generally easier to match, as they are less common than smaller polyps and are more likely to exhibit distinctive features. However, it is essential to note that sessile serrated flat polyps can be challenging for CCE to identify, even when they exceed 10 mm [28,29].

CCE is highly accurate in identifying malignant features, which is helpful in polyp matching, though malignant characteristics may not always be consistently captured from different angles [15,30]. Other features such as vascular pattern, surface details, and contour become important for smaller polyps without prominent morphology. Unique vascular patterns, ulceration, eroded surfaces, or irregular shapes can make a polyp stand out and facilitate matching.

#### 4.1.2. Extra-Polyp Time and Spatial Features for Polyp Matching

To accurately match polyps, we need to carefully review the timestamps of their appearances in the CCE video and examine the section of the video between these timestamps to track the movement of the capsule. This spatial information is important for determining whether the polyps are the same. Additionally, matching is supported by the time interval between the polyp’s appearance on the green and yellow cameras. If the polyp appears within 30 s on both cameras, it is more likely to be the same polyp [25].

When there is insufficient spatial information, other nearby anatomical landmarks can be helpful. These landmarks may include the opening of the appendiceal orifice, ileocaecal valve, anal cushions, hepatic flexure, and splenic flexure. The distance of the polyp from these landmarks can help in identification. However, it has been shown recently that the reliability of flexure landmarks can be inconsistent, which makes matching polyps in these areas less reliable [31]. Without landmarks, other nearby structures, such as sentinel polyps, diverticula, large angioectasia, or lipomas, can serve as relatively reliable markers for polyp matching.

Even with the CCE Polyp Matching criterion, a fundamental limitation is that small sessile polyps, primarily when found nearby or within a short colonic segment, often fail to meet the 5-point matching threshold. They remain challenging to differentiate clinically. In addition, this current score is designed to confirm matches but only allows for the possibility of polyps being the same when the criteria are fully met. Although the weighting of each of the eight components naturally varies depending on the specific polyp types and the observer’s experience, the current system uses equal weight to all factors, which may oversimplify the process. A future scoring system that adjusts the weighting of each component could further refine the polyp matching process and serve as a foundation for developing machine learning models to aid this process in the future.

### 4.2. CCE to Subsequent Colonoscopy Polyp Matching

This issue has been more thoroughly studied in the literature, as colonoscopy is considered the reference standard for assessing CCE accuracy in polyp detection, which naturally requires polyp matching between the two modalities. Multiple studies have consistently shown that more polyps are found in the initial CCE than in subsequent colonoscopy [32,33,34]. This polyp number discrepancy can be attributed to polyps being missed during colonoscopy or imperfections in polyp matching algorithms between the two modalities, resulting in mismatched or incorrectly matched polyps [1]. These manual polyp matching algorithms are often labour-intensive and tedious, particularly when comparing colonoscopy polyp images with those from CCE [1,33,35].

Polyp matching between CCE and colonoscopy is undoubtedly more complex. Our systematic review in this study revealed that no established criteria currently exist for this process. This challenge is particularly relevant in daily practice, as not all colonoscopists are trained to review CCE videos themselves; many rely on the CCE report. This can lead to a loss of critical information, especially when no standard quality assurance framework is in place for CCE reporting. This lack of standardised reporting makes polyp matching more difficult when translating findings from CCE to colonoscopy. Even when the same clinician performs both the CCE and colonoscopy, while this can reduce these challenges, more is needed to eliminate the problem. However, it is critical to ensure that all advanced polyps identified in CCE are detected and removed during colonoscopy. This is particularly important in reducing future legal risks, especially in cases where post-colonoscopy colorectal cancer (PCCRC) develops [36].

Several studies in the literature have indirectly highlighted the challenges in matching polyps between CCE and colonoscopy [32,33,34]. These difficulties primarily stem from inaccuracies in estimating polyp size on CCE and inconsistencies in flexure localisation, which are critical factors for polyp matching. Polyp size has repeatedly been reported as inaccurate and often overestimated in CCE [27,37], partly due to the limitations of the RAPID measuring tools. However, optical colonoscopy is also not entirely reliable in size estimation [38]. As for flexure localisation, there is considerable variation in marking these anatomical landmarks, even among experts demonstrated by Schelde-Olesen et al. [31]. These significant uncertainties make polyp matching unreliable, as these factors are only partially dependable.

Previous studies comparing CCE and colonoscopy accuracy employed overly simplistic methods, using rigid size thresholds that produced binary “matched” or “not matched” [1,27,39] outcomes. To address these limitations, Blanes-Vidal et al. developed a machine-learning algorithm to match polyps across CCE, optical colonoscopy, and histology. Their AI algorithm was designed to match polyps based on size, location, and morphology, which is significant for polyp matching.

**Figure 1:**
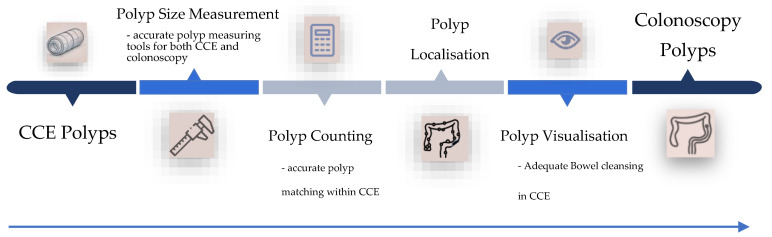
Overview of the Challenges in Matching Polyps from CCE to Colonoscopy.

The algorithm by Blanes-Vidal et al. introduced a more sophisticated approach, quantifying the similarity between polyps using Gower’s Similarity Coefficient (GSC). The GSC provides an average similarity score ranging from 0 (no similarity) to 1 (nearly identical polyps) [10]. Polyp morphology used within the GSC was categorised into five types: pedunculated, broad-based, flat, cancer suspicion, or unknown. The GSC score was calibrated using the dataset’s two extreme polyp features (e.g., the smallest left-sided pedunculated polyp and the largest right-sided non-pedunculated polyp). However, only 168 out of 331 (51%) optical colonoscopy polyps and 375 (45%) CCE polyps met the criteria for successful matching, indicating that approximately 50% of the polyps failed to meet the fundamental matching requirements, mainly due to localisation discrepancies. This issue is less likely intrinsic to the model itself but rather reflects the inaccuracies in polyp localisation and sizing in both CCE and colonoscopy (see Figure 1) [31,40].

Given the uncertainties surrounding polyp size and location in CCE, an approach like the CCE polyp matching criterion might be helpful, particularly by focusing on polyp morphology, vascular pattern, surface characteristics, and contour. However, it is essential to acknowledge that polyp morphology differs between CCE and colonoscopy. Four previous studies consistently noted that flat polyps in colonoscopy often appear polypoidal in CCE [28,29,35,41] (see Appendix A, Table A2). This discrepancy is primarily due to the lack of air insufflation in CCE, unlike in colonoscopy. Therefore, this should be carefully considered when matching polyps in future studies, especially when training machine learning models.

Regarding vascular patterns, surface characteristics, and contour, CCE may offer a different level of detail than colonoscopy, particularly with narrow-band imaging (NBI) capabilities. Although CCE includes technologies such as Flexible Spectral Imaging Colour Enhancement (FICE) and blue mode (BM), FICE is a digital post-processing technique that enhances the visualisation of vascular and mucosal surface patterns compared to using narrow-band optical filters [42]. In optical colonoscopy, however, studies have shown that FICE without magnification does not significantly improve adenoma detection or reduce miss rates compared to white light imaging [43,44].

While FICE in capsule endoscopy may enhance the visibility of pigmented lesions and vascular patterns in CCE, its effectiveness in improving polyp matching between CCE and subsequent colonoscopy has not been specifically studied [45]. Additionally, compared to the gas insufflation used in colonoscopy, the underwater imaging quality in CCE can further affect the ability to assess surface pit patterns. These differences should be accounted for when evaluating polyp characteristics and matching the two modalities.

Another critical factor is the persistent challenge of bowel cleansing quality in CCE. A recent meta-analysis found that the pooled rate of adequate bowel cleansing was 72.5%, with a pooled completion rate of 83% [46]. While introducing prucalopride has improved completion rates, its impact on bowel cleansing quality remains uncertain [4]. Figure 1 highlights the challenges and obstacles that CCE must overcome to achieve accurate polyp matching with conventional endoscopy. Advanced artificial intelligence will need to optimise each step in this process. Combining different AI algorithms may be necessary to achieve reliable polyp matching from capsule to traditional endoscopy.

### 4.3. Driving into the Future

To anticipate future developments, it is essential to reflect on historical patterns of technological evolution. A notable example is the significant technological advancements in Global Positioning Systems (GPS) over the past few decades. The reliance on paper maps for navigation has been replaced by satellite technology and sophisticated machine learning algorithms, enabling real-time location tracking, destination identification, and route optimisation via mobile devices. This technological leap has profoundly impacted daily life and contributed to substantial societal advancement in this area of life.

A similar transformation is currently taking place in the field of CCE. During CCE reporting, polyps in the colon are identified and documented, creating a report that functions as a map. The endoscopist then navigates the colon using this report (map) to locate the polyps for therapeutic interventions or biopsies. However, like early navigation with paper maps, the current limitations in translating CCE findings to conventional colonoscopy can disorient endoscopists. By applying a similar model of satellite navigation evolution to healthcare, the emergence of artificial intelligence in the current CCE field predominantly focuses on the first critical step: polyp detection. A systematic review and meta-analysis by Moen et al. demonstrated that AI algorithms have achieved sensitivity rates of 81.3–98.1% in detecting polyps and colorectal neoplasia, marking an encouraging leap forward for the field [47].

Unfortunately, no CNN-based algorithm has yet been developed to characterise polyps, representing the next logical step in this technological evolution. One key reason for this is the current image quality of CCE, which is insufficient for detailed polyp characterisation [25]. As part of the ongoing efforts to improve polyp matching, enhancing image quality and pathology visualisation as part of the hardware development will be crucial for future advancements. Once these improvements are made, the next foreseeable step for CCE is to develop a system capable of accurately matching polyps detected in CCE with those identified during colonoscopy, addressing the challenges outlined in Figure 1. This innovation would effectively create a “colon GPS”, capable of automatically notifying clinicians which polyps in colonoscopy correspond to those detected in CCE, thus eliminating discrepancies. This approach may require developing an AI system capable of reading CCE images and recording all identified polyps. The same AI could then be applied during subsequent colonoscopy to automatically match and confirm these polyps. A key challenge in this process lies in the stark differences between CCE images and those obtained in a CO2-inflated colonoscopy. However, using a water-submerged insertion technique in colonoscopy might produce images more comparable to CCE, as both are based on a water-submerged rather than air-inflated environment. This could ultimately facilitate AI-automated polyp matching. With this system in place, endoscopists could focus on performing the procedure and therapeutic tasks without the concern of missing or mismatching polyps.

More importantly, a key reason for incorporating AI in this area is to automate the tedious manual process of polyp matching, enabling it to be performed more consistently, efficiently, and continuously. Like the industrialisation of the last century, automating these tasks will allow for scalability, resulting in substantial cost savings. Ultimately, this will result in more efficient and cost-effective gastrointestinal services, addressing the growing demand for colorectal investigations in our already overburdened healthcare systems.

A primary limitation of this study is the limited literature available on this topic. The two direct studies did not specifically examine the false positive rate secondary to duplicate polyp reporting within the same CCE video or its impact on clinical outcomes, such as the need for a follow-up colonoscopy. This limitation made conducting a meta-analysis unfeasible. Additionally, the systematic review was performed by a single reviewer, which increases the risk of missed studies or personal bias in study selection, potentially reducing the comprehensiveness of the review. With advancements in AI on the horizon, proactively refining polyp matching techniques will be essential to align with the evolving standards and skills required for this transformative shift.

## 5. Conclusions

This study underscores that polyp matching in CCE is still relatively new and underdeveloped. Matching polyps within the same CCE video remains a significant challenge, one that will need to be addressed as AI-assisted polyp detection becomes more widely integrated. While ongoing advancements in hardware (imaging technology) and software (artificial intelligence) may help overcome these challenges, this progress is likely still some time away. Moreover, intra-video polyp matching represents only part of the broader challenge of matching polyps between initial CCE and subsequent colonoscopy. Further research is essential to these methods, ultimately elevating CCE as a complementary, non-invasive modality to conventional endoscopy for lower GI investigations.

## Figures and Tables

**Table 2:** Summary of Studies Directly Investigating Polyp Matching Within the Same CCE Video and Between CCE and Subsequent Conventional Endoscopy.

Authors	Year	Study Type	Aspect of Polyp Matching	The Relevant Information to Polyp Matching
Polyp matching within the same CCE video				
Lei et al. [25]	2024	RAND process (modified Delphi process)	All factors considered	Polyp Matching criterion to help match polyps within the same CCE video.
Buijs et al. [24]	2018	Intra- and inter-observer study	Matching polyp number and size within CCE between different readers	Moderate interobserver agreement (experts and beginners) on number of polyps—intraclass correlation coefficient (ICC) = 0.67 (95% CI 0.63–0.83). It improves to 0.70 (95% CI 0.52–0.82) in experts only. High interobserver agreement (experts and beginners) on polyp size measurement—ICC = 0.94 (95% CI 0.78–0.90) but poor interobserver agreement on indication for colonoscopy. Experience contributes to the consistent detection of polyps and suggested a high repeatability for CCE evaluation in experts (with ≥2 years of CCE experience).
Polyp matching between initial CCE and subsequent Conventional Endoscopy				
Blanes-Vidal et al. [26]	2019	Prospective fully paired study	Matching algorithm to match polyp between CCE and colonoscopy	Using machine learning algorithm to match CCE polyps with Optical Colonoscopy (OC) polyps using polyp size, location and/or morphology. Gower’s similarity coefficient (GSC) was used to set threshold by quantifying the similarity between polyp pairs. The proportion of polyps that were assessed as pedunculated was higher by CCE than OC, while the proportion of polyps that were assessed as flat was higher by OC than CCE. Additional polyp features for polyp characterisation (e.g., neoplasia vs. hyperplasia) in CCE were assessed.

**Table 3:** The Colon Capsule Endoscopy Polyp Matching Criteria (CCE PM criteria) [25].

Each Item Is Scored with Equal Weight (1 Point Each)		
Factor Number	Component(s) Within Each Factor	Score
1	Timestamp—within any time interval, consider two polyps as the same if they appear to be so after reviewing the whole section of the colon capsule video between their timestamps.	
2	Localisation—either or: a. Both polyps are within the anatomical landmarks (appendiceal orifice, ileocaecal valve, anal cushions, hepatic flexure and splenic flexure). b. Both polyps are near the same anatomical landmarks.	
3	Vascular pattern—either or: c. Both polyps have similar vascular pattern on the polyp surface only. d. Both polyps have similar vascular pattern interrupted by the polyp.	
4	Polyp size—both polyps are within a 30% size difference.	
5	Time interval of the polyps’ appearance between the green and yellow camera—both polyp images must be within 30 s difference between its appearance between the green and yellow camera.	
6	Surrounding tissue—either or: e. Adjacent small sentinel polyps. f. Adjacent diverticulum/diverticula.	
7	Polyp morphology—one of the following: g. Both polyps have a “pedunculated” appearance. h. Both polyps have a “flat” appearance. i. Both polyps have a “malignant” appearance. j. Both polyps have a “lateral spreading” appearance.	
8	Polyp surface and contour—one of the following: k. Ulceration on both polyps. l. Shape e.g., oval or irregular of both polyps. m. Eroded polyp surfaces on both polyps. n. Distinctive surface colour of both polyps.	
	Total number of score out of 8: (If five or more factors are satisfied during the matching process, it is highly probable that the comparing polyps are the same polyp).	

## Data Availability

The primary data is included in Appendix A, published in the online version of the journal.

## Appendix A

**Table A1:** The Search Term for the Literature search using the PICO framework. These terms were used in combination with Boolean operators and truncations to the search as appropriate in addition to the MeSH terms search. Search limits were not employed due to the concern of missing papers for a comprehensive systematic review.

Domain	Search Terms
Population	Patients, including both paediatric and adult participants, who underwent colon capsule endoscopy (CCE), were found to have polyps, and later underwent a subsequent conventional colonoscopy.
Intervention	Polyp matching techniques or approaches, including algorithms, machine learning models, or scoring systems, designed to reduce duplicate reporting of polyps within CCE and accurately match polyps resected during subsequent conventional endoscopy with those initially identified in CCE.
Comparison	Comparison between CCE readings or CCE-to-subsequent colonoscopy without the use of any of the polyp matching techniques or approaches listed in the intervention.
Outcome	The false positive rate of polyp detection in CCE compared to subsequent colonoscopy after the interventions.

**Table A2:** The Quality Assessment for Diverse Studies (QuADS) of all the selected studies.

Author & Year	Clear Research Concept	Clear Aim	Study Design Justified	Clear Target Population	Sample Strategy	Recruitment Strategy	Data Collection	Format and Content of Data Collection Tool	Data Collection Procedure	Analytic Method Justified	Analysis Appropriateness for the Aim	Research Stakeholders Consideration	Strengths and Limitations Discussed	Score	%
Lei et al., 2024 [25]	3	3	2	2	2	1	2	2	2	2	1	1	2	25/39	64%
Buijs et al., 2018 [24]	3	3	3	2	1	1	2	1	1	3	3	2	2	27/39	69%
Blanes-Vidal et al., 2019 [26]	3	3	3	1	2	1	3	3	3	2	3	1	2	30/39	77%
Semenov et al., 2022 [34]	3	3	1	1	1	0	2	1	1	3	3	2	2	23/39	59%
Spada et al., 2011 [1]	3	3	3	3	3	3	2	3	2	3	3	3	2	36/39	92%
Kobaek-Larsen et al., 2018 [33]	3	3	3	2	2	3	2	2	3	3	3	3	1	33/39	85%
Sakai et al., 2023 [48]	3	3	3	3	3	3	2	2	1	2	3	2	3	33/39	85%
Nakazawa et al., 2021 [49]	3	3	3	1	1	1	2	2	2	3	3	2	2	28/39	72%
Otani et al., 2020 [35]	3	3	1	2	1	1	2	2	1	2	3	1	2	24/39	62%
Yamada et al., 2020 [41]	3	3	1	1	1	1	2	2	1	3	3	2	1	24/39	62%
Utano et al., 2019 [29]	3	3	3	3	3	3	3	3	2	3	3	2	2	36/39	92%
Eliakim et al., 2009 [27]	3	3	3	3	3	3	2	3	3	3	3	2	1	35/39	90%
Eliakim et al., 2006 [50]	3	3	3	3	2	3	2	3	3	3	3	3	2	36/39	92%
Igawa et al., 2017 [28]	3	3	3	3	2	3	2	2	2	3	3	2	2	33/39	85%

**Table A3:** Summary of Studies Highlighting Discrepancies in Polyp Size, Characteristics, Morphology, and Matching Challenges between CCE and subsequent Conventional Endoscopy.

Authors	Year	Study Type	Aspect of Polyp Matching	The Relevant Information to Polyp Matching
Polyp matching between initial CCE and subsequent Conventional Endoscopy				
Semenov et al. [34]	2022	Retrospective study	Size mismatch	False negative rate of CCE (missed lesions in CCE but found in subsequent colonoscopy). In total, 26/184 cases were CCE false negative cases. The number of misclassified CCE lesions based on size alone was 30.8% (8/26). These were more likely due to small polyps which made further matching findings difficult.
Spada et al. [1]	2011	Prospective Trials	Size mismatch	Eighty percent (20/25) of false positive cases with polyps measuring 6–9 mm were because of size mismatching when colonoscopy classified them as <6 mm polyps. Of 7/45 false negative CCE with polyps, 3 were misclassified based on size, which suggested that matching polyps based on size was not reliable.
Kobaek-Larsen et al. [33]	2018	Prospective comparative study	Size mismatch & OC missing polyps	The discrepancy between polyps between CCE and colonoscopy was likely due to false negative findings in colonoscopy due to substantial polyps found in repeat back-to-back colonoscopy. The size discrepancy might be due to an underestimation of polyps in colonoscopy.
Sakai et al. [48]	2023	Prospective trial	Surgical Anastomosis	Nineteen percent (4/21) of anastomotic line were not identified by CCE which might be due to the absence of insufflation.
Nakazawa et al. [49]	2021	Retrospective study	FICE on CCE to characterise polyps	The colour difference (ΔE) between the polyp surface and the surrounding mucosa was calculated using the CIE1976 L*a*b* colour space method on flexible spectral imaging colour enhancement (FICE). This showed an accuracy of 0.928 in differentiating between adenomatous polyps and hyperplastic polyps in CCE.
Otani et al. [35]	2020	Retrospective study	Flat polyp matching	The sensitivity of SSA.Ps was 50% which was not significantly different from protruded polyps, and this is due to their pale colour and location on the right side of the colon where capsule transit time is fast. Forty-one percent (11/27) of superficial colorectal lesions were diagnosed as protruded lesions by CCE.
Yamada et al. [41]	2020	Retrospective study	Flat polyp matching	The sensitivity of detecting flat polyps was 64%, and they appeared polypoidal on CCE.
Utano et al. [29]	2019	Prospective study	Non-polypoid Lateral spreading tumour matching	The sensitivity of CCE for non-polypoid tumours was 87% (4/30 large flat lesions were not detected by CCE (>25 mm) which were located in the proximal colon). Flat large spreading tumours appeared polypoidal in CCE.
Eliakim et al. [27]	2009	Prospective study	Size mismatch Colonoscopy miss polyp	Ten percent (9/98) of detected polyps were 6–9 mm, but they were found to be <6 mm on colonoscopy, and only 3/9 were able to be confirmed visually. A CCE-reported polyp was found to be a haemangioma on colonoscopy. Two cases underwent a second colonoscopy and found the polyps that were missed in the first colonoscopy. This demonstrated that the reason for low specificity of CCE is possibly due to imperfect reference standard colonoscopy and imperfect polyp-matching algorithms, which use polyp size only.
Eliakim et al. [50]	2006	Prospective study	Colonoscopy missed polyp	In total, 3/4 cases where CCE identified significant colonic findings required a second colonoscopy to correctly identify the polyps.
Igwa et al. [28]	2017	Prospective study	Lateral spreading tumour matching	The specificity of CCE in detecting LSTs was 100%, suggesting that they are easy to match due to their large size and morphology. However, LSTs were also missed by CCE in 4/21 cases. It was also reported that the SSA/Ps flat pale polyps were missed in 2 of these cases. In addition, flat LSTs might have appeared polypoidal on CCE. As LST might not be accommodated within a single image, inspection of multiple images might be required to examine the lesions.

The Search strings used in each database:

Pubmed (350)

(capsule endoscope[MeSH Terms] OR capsule endoscopy[MeSH Terms] OR

((capsule*[Title/Abstract] OR videocapsule*[Title/Abstract]) AND (colonoscop*[Title/Abstract] OR endoscop*[Title/Abstract])) OR (capsule*[Text Word] OR videocapsule*[Text Word]) AND (colonoscop*[Text Word] OR endoscop*[Text Word])) AND (colon polyp[MeSH Terms] OR colorectal polyp[MeSH Terms] OR polyp*[Title/Abstract] OR adenoma*[Title/Abstract] OR hyperplastic polyp*[Title/Abstract] OR serrated adenoma*[Title/Abstract] OR (polyp*[Text Word] OR adenoma*[Text Word] OR hyperplastic polyp*[Text Word] OR serrated adenoma*[Text Word] OR polyp match*[Text Word] OR polyp map*[Text Word])) AND (colonoscopy[MeSH Terms] OR large intestine[MeSH Terms] OR ((colonoscop*[Title/Abstract] OR sigmoidoscop*[Title/Abstract]) OR (colonoscop*[Text Word] OR sigmoidoscop*[Text Word])))

Embase (1214)

((capsule endoscop*.mp. or capsule endoscopy/or ((capsule* or videocapsule* or colon capsule* or second-generation capsule*) adj3 (colonoscopy* or endoscopy* or colonic examination* or gastrointestinal examination*)).ti,ab,tw. or (pillcam colon* or (pill adj cam*)).ti,ab,tw. or (pillcam* or (pill adj cam*)).ti,ab,tw.) AND (exp Colonic Polyps/or colorectal polyp/or (polyp* or colorectal polyp* or colonic polyp* or rectal polyp* or sessile polyp* or adenoma* or tubular adenoma* or villous adenoma* or tubulovillous adenoma* or hyperplastic polyp* or serrated adenoma* or polyp match* or polyp map*).ti,ab,tw.) AND

(exp Colonoscopy/or large intestine/or colonoscopy*.ti,ab,tw. or sigmoidoscopy*.ti,ab,tw. or colonic examination*.ti,ab,tw. or gastrointestinal examination*.ti,ab,tw. or bowel examination*.ti,ab,tw. or GI examination*.ti,ab,tw. or lower GI endoscopy.ti,ab,tw. or colonic visualization.ti,ab,tw. or colonic visualisation.ti,ab,tw. or colonic evaluation.ti,ab,tw.))

Medline OVID (250)

((exp capsule endoscopy/or (capsule* or videocapsule*).mp. adj3 (colonoscop* or endoscop*).mp. or (pillcam colon* or (pill adj cam*)).mp. or (pillcam* or (pill adj cam*)).mp.)

AND

((exp colon polyp/or exp colorectal polyp/or polyp*.mp. or adenoma*.mp. or hyperplastic polyp.mp. or serrated adenoma.mp. or (lateral spreading tumour* or lateral spreading tumor*).mp.)

AND

(exp colonoscopy/or exp large intestine/or (colonoscop* or sigmoidoscop* or rectoscop* or anoscop*).mp.)) NOT (duodeno*.mp. or jejeno*.mp. or ileo*.mp.))
